# Involving Parents in Prevention: A Case Example of Research and Intervention Development to Improve Financial Well-Being Support

**DOI:** 10.1007/s10935-026-00910-0

**Published:** 2026-04-04

**Authors:** Nick Axford, Gisella Hanley, Rebecca Summers, Kristin Liabo, Amy Bond, Eleanor Bryant, Georgia Smith, Rachel Silcock, Vashti Berry

**Affiliations:** 1 University of Plymouth, Plymouth, UK; 2 University of Exeter, South Cloisters, St Luke’s Campus, Exeter, EX1 2LU UK; 3 Improving Lives Plymouth, Plymouth, UK

**Keywords:** Community involvement, Co-production, Financial well-being, Lived experience, Parenting, Poverty, Prevention science, Public involvement

## Abstract

The adverse effects on parent and child outcomes of poverty and stressors associated with low income are well established. With notable exceptions, however, the potential role of frontline services in improving family financial well-being is underexplored. This article outlines how patient and public involvement (PPI) was initiated early on in research on financial well-being support (FWbS). It illustrates how research can build on and integrate what parents say about services and how to research them. The involvement work was undertaken in one city in part to inform the adaptation of an income maximisation service to be delivered in local children’s centres. Parents were involved using two approaches: open events with ‘silent conversations’, and individual conversations. Parents were invited to comment on four issues: which families should receive FWbS; how to make FWbS accessible; the process of delivering FWbS; and the nature or content of FWbS. The article describes how we approached parents via existing services, how conversations were facilitated and how parents’ views informed the immediate service redesign and new research on FWbS to improve parent and child outcomes. It also identifies learning, for instance on engaging people not involved in services, involving people throughout a project and offering training and support for researchers and service providers around how to involve people with lived experience of poverty in respectful and meaningful ways.

## Introduction

It is well documented that children who experience poverty have an increased risk of a range of short- and long-term negative health, social, educational and behavioural outcomes (Axford & Berry, 2023). Pathways to these poor outcomes include the adverse effect of low income on parental stress, which affects parenting capacity and skills, and parents’ ability to invest in good nutrition, housing, childcare and educational resources (Carneiro et al., 2025).^1^ Further, experiences of stigma from living in poverty or accessing services for individuals on low income are associated with several aspects of mental health, including negative affect and mental ill-health (Inglis et al., 2023).

Prevention science is dedicated to understanding the causes of poor physical and psychosocial outcomes, using that knowledge to inform policy and practice interventions, and then testing those interventions. Preventive interventions for child psychosocial outcomes tend to focus on enhancing parent–child relationships and parenting skills (e.g., home visiting, parenting support) or child life skills (e.g., youth mentoring), rather than on family financial well-being. This is despite evidence on the adverse effects of poverty and financial stress and studies showing that increasing household income has a positive causal effect on child outcomes, especially for low-income families (Cooper & Stewart, 2021). Possible reasons why prevention science has tended not to address family financial well-being directly include the predominance of psychological theories focused on individual behaviour change, and optimism, with supporting evidence, that the risk of poor outcomes can be mitigated despite inauspicious contexts. There is also a sense that addressing material adversity requires policy or systems change, which is beyond the influence of programmes and individual practitioners.

While policy change is critical to improving financial well-being, for instance around social assistance and employment, frontline services, such as benefits advice integrated in healthcare, can make a difference to family financial well-being and related outcomes (Reece et al., 2022). With notable exceptions, however, such as health visitors linking parents to financial advisors (Johansson et al., 2025; Price et al., 2023), the role of frontline services in improving family financial well-being is underexplored. This is despite many early years, education and social care practitioners having regular contact with families and often being the first port of call for those experiencing financial difficulties. It therefore makes sense for prevention science to consider integrating financial well-being support (FWbS) into universal prevention and targeted early intervention provision for families as a means to improve child outcomes. An essential part of this endeavour is to work with parents to co-produce integrated services and inform related research.

There is a growing movement to involve people with lived experience of poverty in efforts to prevent and address poverty (Broady et al., 2019). For example, Poverty Truth Commissions are initiatives in which people with lived experience of the struggle against poverty work with leaders in a city or region to build a shared understanding of poverty and co-create solutions (Cahill-Ripley & Graham, 2021). This is part of a broader commitment to patient and public involvement (PPI) in service design, with service users understood to be entitled morally to be involved in decisions that will directly impact them or those in similar situations (Smits et al., 2020; Wilson et al., 2015). Historically, and to their detriment, the people most impacted by services such as mental health were not given the opportunity to input into service delivery (Rose, 2013). Recent changes have seen patients and the public being approached to share the valuable experiential knowledge they have gained from engaging with health and social care services (Liabo et al., 2022). Experiential knowledge enables people to identify gaps in service provision unknown to service providers, which helps with reshaping services to ensure that service users’ real needs are identified and met (Liabo et al., 2022; Staley, 2009). Ultimately, PPI has the potential to improve service quality, efficiency and impact (Fredriksson & Tritter, 2017; Thompson et al., 2014). Another intended benefit of PPI is to improve the quality and impact of research. In prevention science, then, PPI might involve maximising the acceptability of an intervention but also optimising procedures for recruiting research participants, collecting data and sharing the results with participants and the wider public (Segrott et al., 2025).

As public involvement in intervention development and research in prevention science advances, there is a need for researchers to report on the design and outcomes of such activity (Segrott et al., 2025). In this article we describe and reflect critically on an opportunity that arose in 2022 in Plymouth, a coastal city in south-west England, to work with parents to consider research around FWbS for families in the context of preventing adverse child outcomes. Plymouth City Council (PCC) funds income maximisation services, such as benefits advice and debt counselling, through Citizens Advice Plymouth (CAP). CAP has a strong track record of providing such services, with quantitative and qualitative evidence of benefits for participants such as increased income and reduced stress. The aim was to build on this good practice under the auspices of PCC’s ‘Community Empowerment Programme (Fair Shares)’ initiative. Specifically, the project involved seeking to adapt income maximisation services to reach low-income families in some of the most deprived communities in the city. The plan was for specialist staff at CAP to support trained volunteers to deliver income maximisation services in accessible community venues, such as children’s centres and food banks.

The first phase involved seeking to co-produce an adapted income maximisation service targeting families, with a view to implementing it in children’s centres in four of the most deprived communities in Plymouth. At the time, income maximisation was delivered remotely by telephone or at the CAP office in the city centre, and involved three levels of increasing intensity to match the problems being addressed: (i) providing information, (ii) offering advice, and (iii) casework. As part of this phase of the project we worked with PCC to engage a range of stakeholders, including parents, to better understand the financial challenges affecting families and how frontline services could be designed to help to address these. This article outlines our approach to working with parents to design service-based FWbS and how it shaped both immediate income maximisation service re-design and our broader prevention research agenda. We consider routes and blockages to parental impact on both, and draw out practice-oriented insights and implications accordingly.

## Co-production Approach to Service Design

**Fig. 1:**
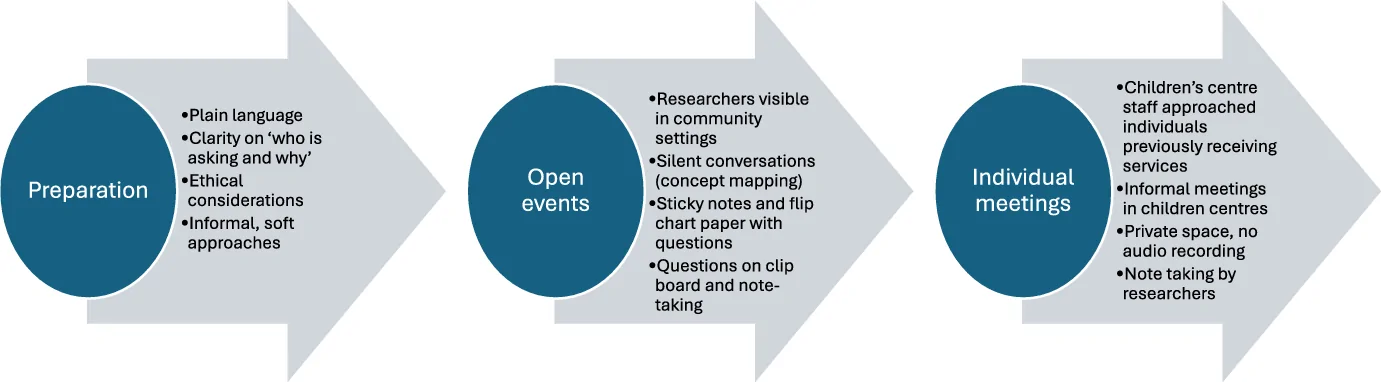
Community-based co-production approach

The work with parents was intended in part to inform the design of the adapted income maximisation service. Researchers met with parents in the period October–November 2022. We followed principles for ethical involvement, including the option of contributing anonymously and freedom to refuse involvement and withdraw at any point (Suri et al., 2024). We also sought to use accessible, non-specialist, language and explained clearly who we were and why we were seeking parents’ views. Research and service design typically happens in professional teams of researchers and service managers. While we could have invited parents to such meetings, we considered it important to seek insight conversations with a broad range of parents in an environment that was familiar to them. Formal research and service meetings can be both intimidating and impractical for parents to attend. To inform service design and the development of future research, we therefore initiated a community-based approach to involvement. Drawing on previous experience and literature on PPI methods (e.g., Hoddinott et al., 2018), two approaches were used to engage parents (conducted by one or more of authors NA, VB and GH): (i) open events and (ii) individual conversations (Fig. 1).

### Open Events

Three events were held at children’s centre venues in the city, each lasting up to two hours and involving an estimated 60–80 parents attending in total. Children’s centre managers advertised the events and facilitated access to locations where we could meet parents in a natural setting: a children’s centre foyer; space at the edge of a large ‘stay and play’ session in a leisure centre; and a room in a community centre adjacent to a community larder. At each event we facilitated ‘silent conversations’ with parents to enable anonymous responses. This collaborative technique is derived from concept mapping (Trochim et al., 1989) and also used in fields such as education and sustainability. It is designed to map a collection of views, allowing participants to respond to what others have written, and preventing louder voices from dominating.

The researchers prepared a set of questions to inform the conversations. Questions centred around: (i) currently available support for addressing family financial issues; (ii) factors that make it easier or harder for families to use such support; and (iii) considerations for the design of a new service (or adaptation of existing services). Parents were also asked about common family financial difficulties or worries, their impact on parents and children, and what effect FWbS services can have on parents and children. Flipchart sheets with the key questions written on them were attached to walls and parents were invited to read others’ responses and to write their responses on sticky notes and stick them on the relevant sheet. Parents were free to discuss the issues with researchers if they wanted to, and researchers facilitated by writing up verbal responses where requested. Refreshments were available during each session. A fourth event involved participating at a pre-arranged ‘fun day’ for children with special educational needs or disabilities (SEND) and their parents. Due to event logistics, we were unable to follow the same format as previous events and instead spoke briefly to families individually, asking the same questions—this time laid out on a clipboard—and making a note of responses. We spoke with 14 families in this way. Participants in open events were not remunerated given the relative brevity of interactions.

### Individual Conversations

We worked with CAP and PCC to identify existing or past service users who were in similar life situations to the intended users of the new service. Managers approached parents from their respective service who had experience of financial difficulties and who would be comfortable discussing financial issues with a researcher. Managers had preliminary conversations with parents to set expectations and ensured that support was available after each conversation as needed. Individual conversations exploring the questions noted above were held with six parents. These lasted approximately 45 min each; two took place in person in children’s centres and four happened over the phone. The researchers took notes with parent permission. Parents taking part in individual conversations received a Privacy Notice beforehand, and in line with PPI guidelines we recognised people’s time to meet with us by offering a £20 multi-shop voucher as a thank you.

### Ensuring Parental Input into Service and Research Design

**Fig. 2:**
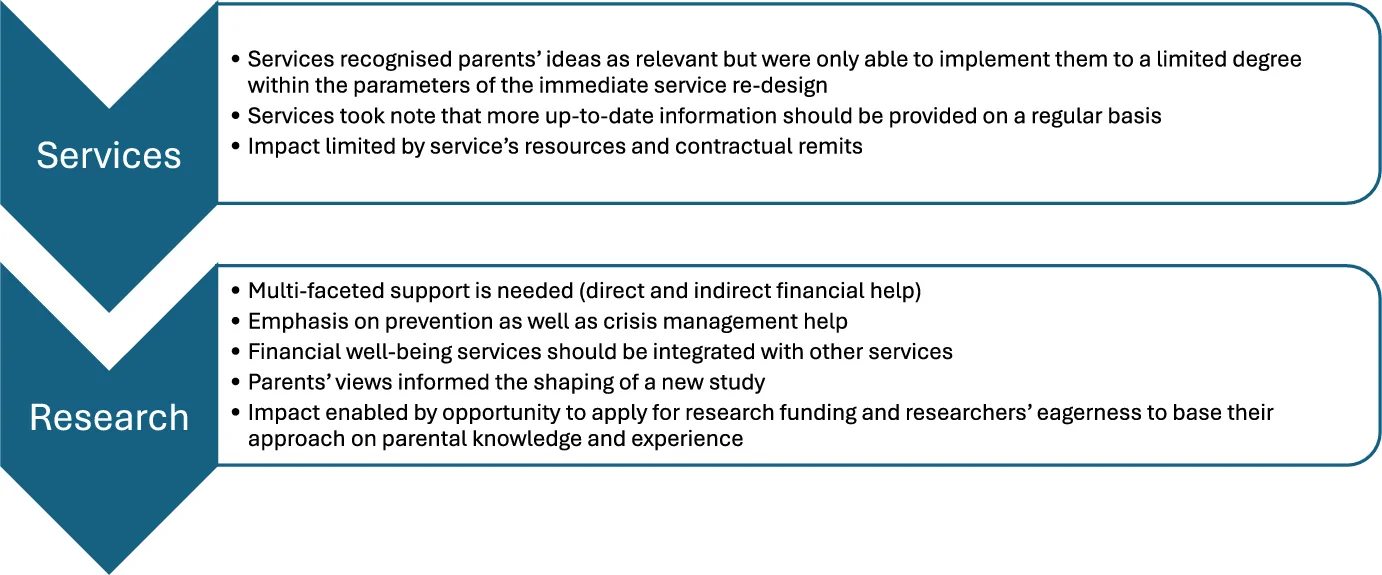
Impact from co-production with parents

After the open events and individual conversations the researchers reflected on the conversations, developed the key points under headings to correspond with our initial questions and started to consider how they could inform the immediate service re-design and our broader research agenda. These points were summarised on a slide set which was then shared in a two-hour online workshop with staff from PCC (n = 3), CAP (n = 2) and children’s centres (n = 4). The workshop focused on implications for (i) a theory of change for how FWbS can contribute to improved child outcomes, and (ii) service design for FWbS provision, including but not limited to the income maximisation service to be implemented in children’s centres. We focus next on how parents’ views informed the adapted income maximisation service and wider research into FWbS for families (Fig. 2).

## The Impact of Parents’ Views

### Impact on Service Design

Several recommendations for adapting the income maximisation service for children’s centres flowed from the views expressed by parents as shared in the workshop. Foremost was the need for children’s centres to emphasise the preventive aspect of the service and frame it positively (e.g., a ‘financial health check’ or ‘money MOT’,^2^ helping families to get their entitlements), rather than restricting it to families thought to be in financial crisis or presenting it as for families who cannot manage their money. Another clear recommendation was that the service should be provided multimodally and that if provided in person in children’s centres (the preferred option) it needs to be in a suitable private space to allow confidential discussions between the Citizens Advice worker and the parent. Additionally, because some parents expressed unease about talking about money to someone they don’t know it was flagged that children’s centre family support workers could support a parent’s attendance and be in the building (if not actually sitting in the session) during an appointment.

That said, there were limits to how much the service could be adapted in line with parents’ views. For example, Citizens Advice were clear that while group sessions could be used to give parents information the more specialised advice work needed to be delivered on an individual basis because it is very specific to a family’s circumstances. Similarly, the range of FWbS that Citizens Advice can offer is limited; it can provide advice on benefits, debt and budgeting but for other types of FWbS families would need to be referred elsewhere. To support this there was an aspiration to produce a regularly updated list of available support. Finally, the idea advocated by some parents of locating FWbS in a range of accessible local community venues could not be acted upon as the decision had already been taken to use children’s centres.

### Impact on Research

The work with parents arguably had a more substantial impact in terms of shaping our long-term research agenda. Parents expressed broadly positive views about the idea of service-level—as opposed to policy-level—FWbS for families. Specifically, the idea of a FWbS service being offered in children’s centres was generally welcomed. Parents offered various examples of FWbS, although it was clear that much of what families had either received or observed other families receiving is fragmented and ad hoc. These experiences indicated that there is an appetite among families to access service-level FWbS but that a clearly thought-through and consolidated offer does not currently exist, suggesting that further intervention development and research is needed to explore feasibility and effectiveness.

As we embarked on further research in this space, the variety of FWbS cited by parents and their articulation of the need for multifaceted support helped to inform our definition of FWbS. Thus, we include the direct provision of money (i.e., cash, vouchers, loans) and goods (e.g., food, clothes, furniture, carpets, white goods, baby items) but also indirect help to manage finances, reduce debt and boost income, whether through help to secure welfare benefits (e.g., help to fill in forms) or support with educational or employment opportunities (e.g., help to find a job or access courses). We also noted the view expressed by parents that there needs to be a preventive angle to FWbS, for instance the idea of offering a financial ‘health check’ rather than waiting until a family reaches crisis point. As such, in our research we are interested in a variety of types of FWbS offered at different levels of the prevention spectrum (i.e., from universal through to treatment), possibly in combination.

Perhaps unsurprisingly given the focus of the project in Plymouth, which involved delivering a FWbS service (income maximisation) in a parenting support setting (children’s centres), parents commented on the potential value of integrating FWbS into other services for parents. Such an approach was generally seen by parents as positive, not least to help facilitate access (e.g., a local community space, going to where parents already are) and reduce stigma but also to enable the more preventive approach advocated by some parents. This idea, and the questions it naturally raises about content and delivery, informed our decision to focus our research primarily on the scope to integrate FWbS with parenting support, notably its feasibility and effectiveness. Our ultimate research goal is to explore the implementation and effectiveness of integrated support. This necessitates understanding different ways in which FWbS and parenting support can be integrated. It also requires potentially developing or optimising an integrated FWbS and parenting support intervention to evaluate in a feasibility study and randomised controlled trial. To prepare the ground for such a project (the ‘main study’) we undertook research in the form of a ‘development study’ to explore several key uncertainties, drawing on our work with parents.^3^  

The first aim was to better understand different models of integrating FWbS with parenting support. This is necessary to inform the sampling frame for the main study. In discussions with parents it was clear that there are various possible models, from being signposted by a children’s centre to a debt relief service, to receiving goods secured by children’s centre staff, to having an outside organisation like Citizens Advice deliver sessions in the children’s centre. It seemed a useful first step to chart these different models and seek to understand their nature, prevalence and strengths and weaknesses according to those involved in delivering them. Thus, in our development study we undertook a national survey of Family Hub managers and commissioners and followed up by interviewing a subset of participants. Family Hubs are funded by the Government and bring together different services for families with children aged 0 to 19 years (or 25 with special educational needs or a disability) in a ‘one stop shop’. They have been rolled out across England, in many cases replacing children’s centres. We were particularly interested in referral pathways to receive FWbS, partnerships between Family Hubs and local or national debt or welfare advice organisations, levels of integration (system, service, programme, individual), the nature of any FWbS already provided by Family Hubs and how it is provided, and which (if any) Family Hub staff had designated responsibility to support families’ financial well-being.

A second uncertainty concerned what is suitable content of FWbS when integrated with parenting support. Understanding more about this before the main study is necessary to inform intervention design or optimisation. The parents we spoke with in Plymouth emphasised the need to tailor such support to a family’s circumstances and needs, particularly taking into account the root of any financial difficulties as this affects what support may be offered (e.g., around a child’s special educational needs). They also mentioned parents’ desire to have opportunities to offer as well as receive, and the potential value of support around finances for children and young people, for instance on understanding the cost of living. As part of the development study we therefore decided to conduct focus groups on these and other issues regarding content with debt and welfare advisors and the developers and purveyors of parenting programmes. We were particularly interested in what they considered acceptable and feasible in terms of offering FWbS in the context of parenting support. We additionally undertook a systematic scoping review of the international literature to better understand the design and content of existing programmes that integrate FWbS and parenting support, including the nature of FWbS and parenting support respectively and if and how they are connected. Prior to the work with parents we had assumed that FWbS would need to be delivered alongside parenting support but through that work we came to appreciate the potential value of this more integrated within-programme approach.

The third set of issues arising in our PPI work with parents in Plymouth and subsequently researched in the development study concerns how best to deliver FWbS to families, especially in a parenting support context. Again, we need to understand this to inform intervention design or optimisation for the main study. The views of parents in Plymouth indicated a need for flexibility of format, delivery modes, timing, providers and settings to address differing parental needs and preferences. Parents also stressed the need to attend to practical and emotional barriers to parents accessing FWbS, especially given possible sensitivities around family finances and the additional costs involved for some forms of access. All data collection with Family Hub staff, debt/welfare advisors and parenting programme developers or purveyors in the development study addresses these topics. It has allowed us to explore what works well and what the challenges are, and to tease out specific issues raised by parents, such as how best to make parents aware of support and reduce stigma associated with accessing FWbS. We have also been able to investigate the perceived acceptability and feasibility from different perspectives of delivery options where parents expressed contrasting views, such as in-person vs. text, phone or online, group vs. individual, and working with a trusted practitioner known to the parent vs. someone they don’t know (to afford privacy and avoid embarrassment).

## Lessons and Implications

There is comparatively little published literature on what parents want from FWbS or how parents’ views on this have shaped prevention practice or research. More broadly, the detail of how PPI activity has informed research design is seldom reported. We have sought to address these gaps by reporting how we sought to bring the views of parents into service design and early study design discussions around FWbS. We have detailed how this PPI activity shaped a specific service re-design process (to a limited degree) and broader prevention research agenda (to a larger degree). While there is a growing body of qualitative research asking parents’ their views on financial well-being and service provision (e.g., Johansson et al., 2025; Price et al., 2023), it was important for the study team to consult with parents directly. This enabled us to learn from parents in an unfiltered way—not through researchers’ analysis of parents’ views, but directly from parents themselves and with the opportunity to ask follow-up and clarifying questions.

Some aspects of the PPI worked well. It was inclusive in terms of involving a significant number of parents, including those from low-income groups and parents of children with SEND. All events and conversations took place where parents were naturally present, and input from parents in individual conversations was recognised financially. The large number of parents engaged in open sessions arguably reflects several factors: they were held in natural settings for parents, including transitional spaces such as a children’s centre lobby; they were advertised and endorsed by centre managers and parenting practitioners; we offered a range of refreshments appealing to adults and children; and we sought to adopt an open, friendly and flexible approach. We were open with parents about the purpose of the work, both in writing and verbally, and reported their views faithfully to the group overseeing the income maximisation service redesign for children’s centres.

We are also aware that our PPI work had limitations, however, with learning to be gained. The parents consulted were already accessing parenting support or FWbS services, and in some cases were encouraged by children’s centre and CAP staff to participate, so the views expressed may not fully reflect those of parents who have *not* engaged with those services. It might also mean that parents with conflicting views or challenging behaviour were not invited. This is a reoccurring conundrum for PPI in prevention research; research teams want to understand people’s experiences of not engaging with preventive interventions but the people not engaging in such provision are unlikely to engage in PPI activities. There may be lessons for addressing this from research on how to engage disadvantaged or vulnerable parents in services, for example through outreach and systematically addressing practical and emotional barriers to participation (Pote et al., 2019). Scope for making changes to the income maximisation service for children’s centres based on PPI was limited by set aspects of the service, including licensed content and the need for delivery by trained CAP advisors or volunteers, and by decisions already taken regarding the adaptation. Parents were brought in part way through the project, restricting their ability to shape the service more fundamentally, and they were not involved in summarising key points or present at the meeting where their views were represented. Involving parents earlier and throughout may have helped mitigate this.

It is widely acknowledged that PPI with marginalised communities requires adequate time and resourcing to allow for building relationships that support open communication with iterative feedback and supporting participants to develop new skills and connect with others (Liabo et al., 2020; Segrott et al., 2025). This also needs proper administration to facilitate practicalities, such as scheduling meetings and travel. Although the aforementioned limitations reflect the resources available for what was effectively a pump-priming project, the development study informed by the work reported here had a group of parents who met monthly to input on research decisions throughout the project. This group was facilitated by a member of an experienced involvement team which works to a PPI strategy aligned to National Institute for Health and Care Research (NIHR) Public Involvement Standards.^4^ While adequate resourcing goes a long way to addressing engagement challenges, there are particular issues that can arise in this kind of work that need to be worked through sensitively with appropriate training and support. These include nervousness around working with people with lived experience of poverty and who might not have the same educational background as those facilitating the engagement, and the way in which researcher eagerness to not stigmatise can unintentionally exclude some people (Liabo et al., 2017). It is exactly to counter this that we have outlined an approach that can act as a first step towards the deeper involvement of parents in a sensitive area of research, where they have much knowledge and insight to offer.

## Footnotes

1 The term ‘parent’ is used throughout the article to denote a child’s primary carer(s).

2 A Ministry of Transport (MOT) test is an annual legal vehicle safety inspection in the UK. The term ‘MOT’ is used colloquially to denote a check-up, for example of a person’s health.

3 The development study is described in more detail here: https://doi.org/10.3310/nihropenres.1115272.1.

4 https://sites.google.com/nihr.ac.uk/pi-standards/home.
